# Efficacy and safety of Shexiang Baoxin Pill combined with Western medicine in the treatment of acute myocardial infarction: A single-center, double-blind, randomized controlled trial

**DOI:** 10.1097/MD.0000000000024246

**Published:** 2021-01-22

**Authors:** Yan Yang, Songtao Gao, Qiuju Fang, Mei Zhu

**Affiliations:** Department of Cardiovascular Medicine, Heilongjiang Provincial Hospital, Harbin 150001, Heilongjiang Province, China.

**Keywords:** acute myocardial infarction, combined therapy, protocol, randomized controlled trial, Shexiang Baoxin Pill

## Abstract

**Background:**

The morbidity and mortality of acute myocardial infarction are on the rise, and the efficacy of conventional treatment is limited. Shexiang Baoxin Pill is a kind of proprietary Chinese medicine, which has been widely used in the treatment of acute myocardial infarction in China, and has certain advantages. At present, there is a lack of strict randomized controlled trials to verify the efficacy and safety of Shexiang Baoxin Pill combined with Western medicine in the treatment of acute myocardial infarction. Therefore, the purpose of this randomized controlled trial is to evaluate the clinical efficacy of Shexiang Baoxin Pill combined with Western medicine in the treatment of acute myocardial infarction.

**Methods:**

This is a prospective randomized controlled trial to study the efficacy and safety of Shexiang Baoxin Pill combined with Western medicine in the treatment of acute myocardial infarction. It is approved by the Clinical Research Society of our hospital. According to 1:1, the patients will be randomly divided into observation group (Shexiang Baoxin Pill combined with Western medicine group) and control group (routine Western medicine group). The patients in the 2 groups will be treated continuously for 4 weeks and followed up for 3 months. Pay attention to its curative effect index and safety index. The observation indexes included total effective rate of improvement of cardiac function, left ventricular ejection fraction (LVEF), endothelin (ET), nitric oxide (NO) level, interleukin-6 (IL--6), adverse reactions, and so on. We will analyze the structure by SPSS version 19.0.

**Discussion:**

This study will evaluate the efficacy and safety of Shexiang Baoxin Pill combined with Western medicine in the treatment of acute myocardial infarction. The results of this experiment will provide clinical basis for Shexiang Baoxin Pill combined with Western medicine in the treatment of acute myocardial infarction.

**Trial registration:**

OSF Registration number: DOI 10.17605/OSF.IO/PYJTK.

## 1 Introduction

Acute myocardial infarction (AMI) refers to a sharp decrease or complete interruption of coronary artery blood supply caused by various reasons, resulting in serious and lasting acute myocardial ischemia leading to cardiomyocyte necrosis.^[1]^ It is the most serious manifestation of coronary artery disease and is also one of the main causes of death in patients with coronary heart disease.^[2]^ With the change of lifestyle and the increasing degree of population aging, its morbidity and mortality are increasing year by year, bringing a heavy burden to individuals, families, and society.^[3]^ Typical clinical manifestations are severe pain in the precordial region, suffocation with sweating, and pain is not easy to relieve, can be complicated with cardiogenic shock, arrhythmia or heart failure, the disease develops rapidly, often life-threatening, and the prognosis is poor.^[4]^ Therefore, timely and effective treatment of AMI is the key to reduce the mortality and disability rate.

The morbidity and mortality of AMI are increasing year by year, and percutaneous coronary intervention (PCI) is an effective treatment for the disease. In addition, it also includes thrombolysis, anticoagulation, anti-platelet aggregation, improving ventricular rate, preventing ventricular remodeling, and enhancing myocardial energy metabolism.^[5,6]^ At the same time, traditional Chinese medicine also plays an important role in its prevention and treatment.^[7]^ Shexiang Baoxin Pill is a traditional Chinese medicine preparation commonly used in the clinical treatment of AMI in China. In 1993, it was listed as one of the emergency essential drugs by the State Administration of traditional Chinese Medicine.^[8]^ Shexiang Baoxin Pill is also recommended for the treatment of AMI patients in the “consensus of experts on diagnosis and treatment of Acute Myocardial Infarction with Integrated traditional Chinese and Western Medicine” issued by the Cardiovascular Professional Committee of Chinese Society of Integrated traditional Chinese and Western Medicine in 2018. Shexiang Baoxin Pill is composed of artificial musk, Storesin, toad, artificial bezoar, cinnamon, borneol, and ginseng extract, which can relieve chest pain, inhibit platelet aggregation in patients with AMI, reduce blood lipids, improve vascular endothelium and cardiac function, and has good safety.^[9]^ At present, it has been proved that it has satisfactory clinical efficacy and high safety in the treatment of cardiovascular diseases such as chronic heart failure, coronary heart disease and acute coronary syndrome.^[10–13]^ Although Shexiang Baoxin Pill has been used in clinic, there is still a lack of strict randomized controlled trials to study the efficacy and safety of Shexiang Baoxin Pill combined with Western medicine in the treatment of myocardial infarction. Therefore, we intend to use this randomized controlled trial to evaluate the clinical efficacy of Shexiang Baoxin Pill combined with Western medicine in the treatment of AMI.

## 2 Materials and methods

### 2.1 Study design

**Figure 1:**
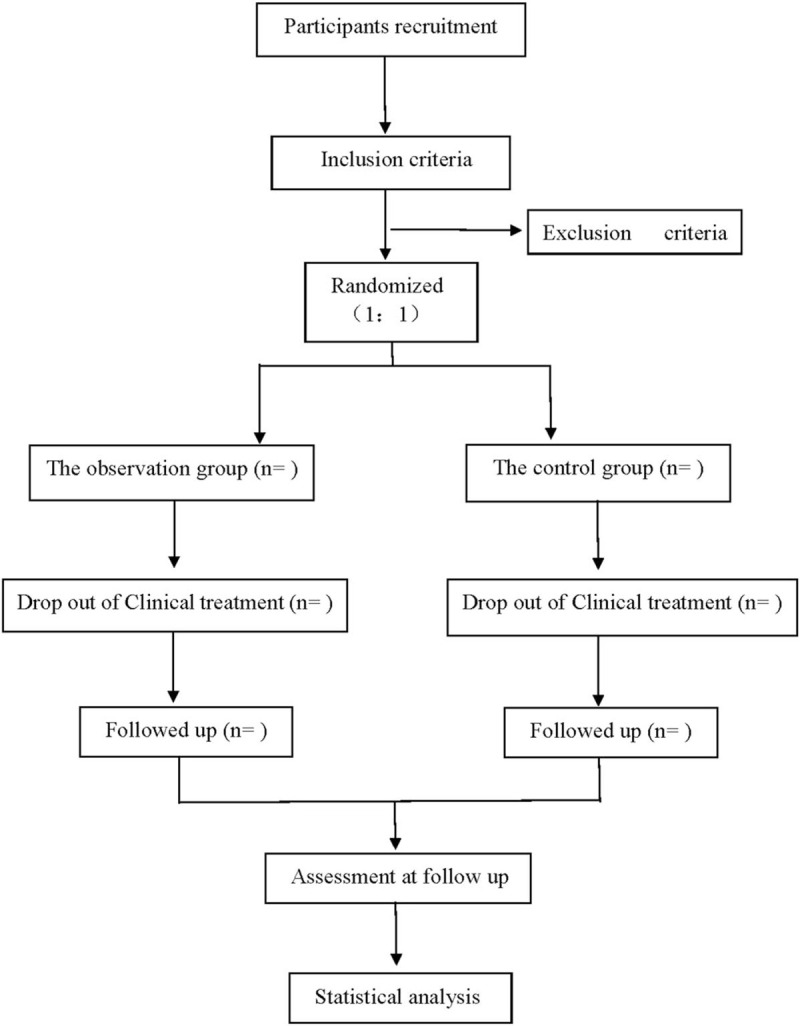
Flow diagram.

This is a prospective, double-blind randomized controlled trial to study the efficacy and safety of Shexiang Baoxin Pill combined with Western medicine in the treatment of AMI. This experiment will follow the comprehensive trial reporting standard.^[14]^ The flow chart is shown in Figure 1.

### 2.2 Ethics and registration

This research scheme is in line with the Helsinki Declaration and approved by the Clinical Research Ethics Committee of our hospital. This laboratory is already registered in the open science framework (OSF) (registration number: DOI 10.17605/OSF.IO/PYJTK). Before randomly grouping, all patients are required to sign a written informed consent form. Participants can withdraw from the study at any time for any reason. After withdrawal, the patient's data will be used with the patient's consent.

### 2.3 Patients

(1) Inclusion criteria: ① meet the diagnostic criteria of AMI, and diagnosed by clinical manifestations, myocardial markers, electrocardiogram and other auxiliary examination; ② onset time is within 24 hours, taking nitrate drugs cannot be relieved; ③ the age is 30 to 80 years; ④ there is no Shexiang Baoxin pill and other related drugs within 3 months before admission; ⑤ voluntary subjects, sign informed consent, good compliance.
(2) Exclusion criteria: ① patients with heart failure induced by other diseases such as congenital heart disease; ② patients with malignant tumors or other liver, kidney, and lung diseases; ③ patients with other heart diseases such as severe arrhythmia; ④patients with severe mental illness; ⑤ patients with allergies or contraindications to medication in this study.

### 2.4 Randomization and blinding

Randomly grouped by independent statisticians, random sequences are generated using SAS V.9.4 software, and the numbers are stored in opaque envelopes. One hundred twenty patients will be randomly divided into observation group and treatment group according to the rate of 1:1. The allocation of research by attending physicians, patients, assistant researchers, data statistical analysts, and nurses is unknown throughout the study.

### 2.5 Study design

After admission, patients in both groups will be given routine treatment, and electrocardiogram, blood pressure and blood oxygen saturation will continuously be monitored. Each patient inhaled oxygen with low flow for 3 days, and each patient will be told to rest in bed according to his or her own condition. Seventy-five milligram, qd of hydroxyclopidogrel sulfate, 100 mg, qd of aspirin enteric-coated tablets and 20 mg, qn of Atto vastatin calcium tablets will be taken orally.

The observation group will be treated with 67.5 mg, tid of Shexiang Baoxin Pill on the basis of routine treatment, while the control group will be treated with placebo instead of Shexiang Baoxin Pill. The appearance, texture, and smell of the placebo will be consistent with those of Shexiang Baoxin Pill. The attending physician can adjust the medication according to the specific conditions of the patient, and all schemes will be recorded in detail for final result analysis.

During the treatment period, the distribution of drugs will be distributed to the corresponding numbered patients by nurses who do not know about the grouping according to the drug number.

### 2.6 Evaluation criteria and efficacy judgment

(1) Main outcome indicators: ①Total effective rate of improvement of cardiac function (refer to the principle of clinical research on treating heart failure with new Chinese Medicine),^[15]^ effective: ECG is stable, clinical symptoms basically disappear; improvement: ECG is basically stable, clinical symptoms are improved; ineffective: clinical symptoms, ECG do not change or aggravate.
(2) Secondary outcome index: left ventricular ejection fraction (LVEF), endothelin (ET), nitric oxide (NO) level, interleukin-6 (IL--6), C-reactive protein (CRP).
(3) Adverse reactions: including abnormal liver and kidney function and any uncomfortable symptoms during treatment (such as arrhythmia, digestive tract reaction, etc).

### 2.7 Data collection and management

The data will be collected according to the evaluation criteria before the beginning of treatment, 72 hours after the beginning of treatment and at the end of treatment. Each patient will be followed up by outpatient or telephone within 3 months after the end of treatment, and the reasons for the loss of follow-up cannot be collected in detail. All data will be collected jointly by 1 or 2 assistants. Personal information about potential participants and registered participants will be collected, shared, and stored in a separate storeroom to protect before, during, and after the test confidentiality. Access to the database is limited to the researchers of this research group.

### 2.8 Statistical analysis

The data will be processed by SPSS19.0 statistical software, the measurement data will be expressed by $\bar{x}\pm s$, *t* test, counting data will be expressed by rate (%), and *χ*^2^ test will be used. When *P <* .05, the difference is statistically significant.

## 3 Discussion

In recent years, the incidence of AMI has been younger, and the overall mortality has been increasing. Its morbidity and mortality are the first in cardiovascular disease.^[16]^ At present, the main surgical treatments are thrombolysis and PCI, including β-blocker, angiotensin-converting enzyme inhibitor (ACEI), angiotensin receptor antagonist, statins, antiplatelet drugs, receptor antagonists, and aldosterone antagonists. Although the above treatment improved the prognosis of patients, it did not reduce the mortality.^[17]^ Beta-blockers have no short-term effect on mortality and may increase the risk of cardiogenic shock; thrombolytic agents can relieve symptoms but may also lead to stroke and massive bleeding; 10% to 18% of coronary heart disease survivors suffer from secondary myocardial infarction, stroke, or cardiovascular death after PCI.^[18,19]^

Traditional Chinese medicine has been widely used in the treatment of AMI for hundreds of years. Many studies have shown that a single traditional Chinese medicine or compound preparation has a variety of targeted effects in the prevention and treatment of cardiovascular diseases.^[20,21]^ Shexiang Baoxin Pill has been used in the treatment of cardiovascular disease in China for more than 30 years, and achieved good results.^[22]^ Modern pharmacological studies suggest that Shexiang Baoxin Pill has the effects of anti-inflammation, antioxidation, improving endothelial cell function, relieving vascular inflammation, stabilizing vascular plaque, promoting angiogenesis, and anti-fibrosis by reducing plasma C-reactive protein. It can significantly improve the oxidative stress injury of cardiomyocytes due to ischemia and hypoxia and reduce the area of myocardial infarction.^[13,23]^ Animal experiments show that Shexiang Baoxin Pill can protect myocardium by regulating lipid metabolism and improving cell mitochondrial function.^[24]^ By activating macrophages and releasing pro-angiogenic factors such as Vegf-a, Shexiang Baoxin Pill can significantly increase the expression of mRNA and protein related to angiogenesis, promote angiogenesis,^[25]^ and achieve the effect of anti-angiosclerosis by improving inflammatory reaction and inhibiting lipid accumulation.^[26]^ A lot of evidences have shown that Shexiang Baoxin Pill has advantages in the treatment of AMI, but there is a lack of strict clinical studies. Therefore, this randomized controlled trial will explore the efficacy and safety of Shexiang Baoxin pill combined with conventional Western medicine in the treatment of AMI, and provide a new scheme for the treatment of AMI.

This study also has some limitations: due to the short planned follow-up time, we are unable to understand the impact of long-term results, so we may extend the follow-up time if necessary; this study is a single-center study, including patients may have regionalization, which has a certain impact on the results.

## Author contributions

**Data collection:** Yan Yang and Songtao Gao.

**Funding support:** Mei Zhu.

**Investigation:** Songtao Gao.

**Resources:** Songtao Gao, Qiuju Fang.

**Software operating:** Qiuju Fang.

**Supervision:** Qiuju Fang and Mei Zhu.

**Writing – original draft:** Yan Yang, Songtao Gao.

**Writing – review & editing:** Yan Yang, Mei Zhu.
